# Oral microbiome test as an alternative diagnostic tool for gastric alterations: A prospective, bicentric cross-sectional study

**DOI:** 10.1371/journal.pone.0314660

**Published:** 2024-12-02

**Authors:** Fernanda Prata Martins, Jessica Andrade-Silva, Bianca Luise Teixeira, Angelo Ferrari, Ana Paula Christoff, Giuliano Netto Flores Cruz, Fernanda Vieira Paladino, Luiz Felipe Valter de Oliveira, Camila Hernandes

**Affiliations:** 1 Hospital Israelita Albert Einstein, Sao Paulo, São Paulo, Brazil; 2 BiomeHub Biotechnologies, Florianópolis, Santa Catarina, Brazil; Defense Threat Reduction Agency, UNITED STATES OF AMERICA

## Abstract

The human microbiome plays a pivotal role in influencing various physiological processes and maintaining overall well-being, including the gastric system. Current diagnostic tests for gastric diseases often involve invasive procedures, sampling limitations, and medication effects, leading to potential diagnostic errors and discomfort to patients. Considering the connection between oral and gastric microbiomes, this cross-sectional study aimed to assess the diagnostic potential of the oral bacterial profile in patients undergoing upper digestive endoscopy. Oral samples from 266 participants across two Brazilian sites (Belterra and Sao Paulo) were sequenced and subjected to bioinformatic analysis to identify microbiome composition across endoscopy outcome groups, exploring alpha and beta-diversity, richness, and differential abundance and prevalence. *Prevotella*, *Haemophilus*, *Fusobacterium*, *Neisseria*, and *Streptococcus* were the most abundant genera observed. No significant associations were found between alpha diversity profiles and endoscopy outcomes; beta diversity analyses similarly showed no correlations. Overall, the study did not establish the oral microbiome as a reliable marker for gastric health, underscoring the necessity for broader studies in the development of non-invasive diagnostic tests.

## Introduction

Various regions of the body harbor distinct microbial populations, each contributing to specific functions and interactions. The balance of bacterial composition, metabolic activities, and distribution within the gut plays a crucial role in determining overall health and susceptibility to illness. The relationship between oral and gastric microbiota is intricate, dynamic, and interconnected, with a complex interplay between these microbial communities that can influence oral and gastric health [1].

The global prevalence of upper digestive system disease cases was 780.59 million in 2019 [2], representing about 10% of the world’s population; changes in the structure and function of the stomach can be caused by the presence of microorganisms or the imbalance of the local microbiota. *Helicobacter pylori*, a gram-negative pathogen, is the predominant microorganism associated with gastric infections, with approximately half of the world’s population being colonized [3]. *H*. *pylori* significantly increases the risk of chronic gastritis, ulcers, and various forms of cancer, including adenocarcinoma, as established by World Health Organization’s International Agency for Research on Cancer (IARC). Other microbes than *H*. *pylori* also play a role in the development of gastric cancer, such as *Peptostreptococcus*, *Desulfovibrio*, and *Fusobacterium* [4,5]. Changes in the gastric microbial structure could be seen in different precancerous stages, from superficial gastritis to atrophic gastritis, and gastric intraepithelial neoplasia to gastric cancer [6]. In that sense, the gastric microbiota might play different roles in carcinogenesis.

Multiple invasive and non-invasive diagnostic tests are available to detect pathogenic microorganisms that can potentially influence human health. The selection of a suitable test depends on factors such as test availability, the patient’s clinical condition, and the diagnostic accuracy observed in different clinical scenarios. Invasive procedures, like upper digestive endoscopy, are commonly employed to diagnose *H*. *pylori* infection. Additional invasive tests can be performed on the mucosal tissue by obtaining a gastric biopsy, including urease testing, histology, culture, and molecular methods [7]. However, since the distribution of infection within the gastric mucosa is not uniform, there is a possibility of diagnostic errors due to sampling limitations. Furthermore, certain medications like proton pump inhibitors and antibiotics can decrease the sensitivity of those tests [8–10].

Considering the vital link between the oral and gastric microbiome, previous studies have shown that changes in the richness, evenness, and/or number of bacterial species inside the oral cavity could be a diagnostic biomarker for chronic gastritis [11] and gastric cancer [12,13].

Therefore, developing a non-invasive method that evaluates the oral microbiome from a buccal sample seems important for gastric disease screening and diagnosis. In this study, we aimed to evaluate the oral bacterial profile in patients referred for upper digestive endoscopy to assess its diagnostic potential related to gastric alterations.

## Methods

### Ethics statement

The study was approved by the Research Ethics Committee of Hospital Israelita Albert Einstein (approval numbers 2.392.780 and 4.333.608) and was conducted according to the principles expressed in the Declaration of Helsinki. All subjects provided written informed consent.

### Study design and setting

A prospective cross-sectional survey was carried out at two centers in Brazil, located in Belterra (Cohort 1) and São Paulo (Hospital Israelita Albert Einstein—Cohort 2) from November to December 2017, and from March to September 2021, respectively. The primary outcome was a composite of gastric alterations as detected in endoscopy, including erosive esophagitis and/or gastroduodenal peptic disease. The study is reported according to both the STROBE statement for cross-sectional investigations and the STORMS statement for human microbiome studies.

### Participants

Participants with an indication of upper digestive endoscopy to detect gastric alterations were recruited consecutively based on predefined inclusion and exclusion criteria. The study inclusion criteria were age > 18 years old, referral to upper digestive endoscopy for detection of gastric alterations, even when the participants were using proton pump inhibitors and/or antibiotics. Participants who had eaten or brushed their teeth in the 4 hours before the sample collection were excluded. Also, we excluded participants with esophagus, stomach, or duodenum surgery history since this factor can change the endoscopic findings regardless of the presence of microorganisms. Those who did not sign the informed consent form were not included.

### Sociodemographic, lifestyle, and clinical variables

After consent, all eligible participants answered a specific questionnaire used for sociodemographic, lifestyle, and clinical data collection. Sociodemographic variables included age, sex, and education; lifestyle habits data included smoking and alcohol use. Clinical variables consisted of symptoms, use of proton pump inhibitors, use of antibiotics in the last 30 days, and the upper digestive endoscopy results.

### Sample collection, DNA extraction, and 16S rRNA amplicon sequencing

For oral microbiome analysis, sampling was performed using sterile nylon flocked swabs (Copan Inc., Italy or Puritan, USA) and stored in a stabilizing solution (BiomeHub, Brazil) for transport at room temperature. Samples were collected with a swab from the posterior region of the oral cavity (which includes teeth (molars and premolars), plaque regions, oral mucosa, and dorsal tongue). This approach covers multiple oral sites and comprehensively represents the oral microbiota, reducing potential bias from specific areas. The swabs were transported to the laboratory facilities at room temperature and processed within a maximum of 30 days after sample collection. Bacterial DNA from the samples was obtained using the DNeasy Qiaamp DNA Blood Mini Kit (QIAGEN) according to the manufacturer’s instructions.

Amplicon sequencing library preparation for bacteria was performed using the V3/V4 16S rRNA gene primers 341F (CCTACGGGRSGCAGCAG) [14] and 806R (GGACTACHVGGGTWTCTAAT) [15] in a two-step equivolumetric PCR protocol [16]. The first PCR was performed with V3/V4 universal primers containing a partial Illumina adaptor, based on TruSeq structure (Illumina, USA), allowing the second PCR with indexing sequences. The PCR reactions were carried out in triplicates using Platinum Taq (Invitrogen, USA) with the conditions: 95°C for 5 min, 25 cycles of 95°C for 45s, 55°C for 30s and 72°C for 45s, and a final extension of 72°C for 2 min for PCR 1. In PCR 2, the conditions were 95°C for 5 min, 10 cycles of 95°C for 45s, 66°C for 30s and 72°C for 45s, and a final extension of 72°C for 2 min. The final PCR reactions were cleaned up using AMPureXP beads (Beckman Coulter, USA) and an equivalent volume of each sample was added in the sequencing pool. Negative control reactions were included to assess possible PCR reagent contaminations. The DNA concentration of the libraries pool was estimated with Picogreen dsDNA assays (Invitrogen, Waltham, MA, USA) and then diluted for accurate qPCR quantification using the Collibri Library Quantification kit (Invitrogen, USA). The sequencing pool was adjusted to a final concentration of 11 pM and sequenced in a MiSeq system (Illumina, USA), using the standard Illumina primers provided by the manufacturer kit. Single-end 300 cycle runs were performed using a V2 × 300 sequencing kit (Illumina, USA) with average sample coverages set to 45,000 reads per sample in all sequencing runs.

### Bioinformatics analysis

The read sequences were analyzed using a bioinformatics pipeline previously described [16–18] (BiomeHub, Brazil-hospital_microbiome_rrna16s: v1). Illumina FASTQ files had the primers trimmed and their accumulated error evaluated [16]. Reads were analyzed with the Deblur package [19] to remove possible erroneous reads and then identical read sequences were grouped into oligotypes (clusters with 100% identity, ASVs amplicon sequencing variants). Next, VSEARCH[20] was used to remove chimeric amplicons. An additional filter was implemented to remove oligotypes below the frequency cutoff of 0.2% in the final sample counts. We also implemented a negative control filter since oral microbiomes generally are low biomass samples [16]. In each processing batch, we used negative controls (reagent blanks) for the DNA extraction and PCR reactions. If any oligotype is recovered in the negative control results, they are checked against the samples and automatically removed from the results only if their abundance (in number of reads) are no greater than two times their respective counts in the sample. The remaining oligotypes in the samples are used for taxonomic assignment with the BLAST tool [21] against a reference genomic database (encoderef16s_rev6_190325). This reference database was constructed with complete and draft bacterial genomes, which were obtained from NCBI, focused on clinically relevant bacteria. It is composed of 11,750 sequences, including 1,843 different bacterial taxonomies.

Taxonomy was assigned to each oligotype using a lowest common ancestor (LCA) algorithm. If more than one reference can be assigned to the same oligotype with equivalent similarity and coverage metrics, the taxonomic assignment algorithm leads the taxonomy to the lowest level of possible unambiguous resolution (genus, family, order, class, phylum, or kingdom), according to the similarity thresholds [22].

### Statistical analysis

All statistical analyses were carried out using R (v. 4.0.2). Baseline characteristics were summarized as median and inter-quartiles range or as absolute frequency and percentages, stratified by the primary outcome. Alpha- and beta-diversity analyses employed the Shannon index and the Bray-Curtis dissimilarity, respectively. Beta-diversity was presented as Principal Coordinate Analysis with PERMANOVA marginal tests using the adonis2 function from the vegan package (v. 2.6.4) [23]. We also assessed richness as a secondary alpha-diversity outcome. Differential abundance (DA) analysis employed four methods to reach consensus: corncob (v. 0.2.0), DESeq2 (v. 1.36.0), limma+voom (3.52.0), and linDA/MicrobiomeStat (v. 1.1) [24–27]. We also added a fifth method to test differential prevalence, i.e., the likelihood of binary detection of each taxon across study groups (or “presence/absence analysis”). Differential prevalence (DP) was assessed using Firth’s Bias-Reduced Logistic Regression as implemented in the logistf R package (v 1.24.1) [28]. False-discovery rate (FDR) was controlled at 10% for the DA/DP analyses using the Benjamini-Hochberg procedure [29]. FDR correction was performed within each DA/DP tool for each taxonomic rank (including amplicon sequencing variant, species, genus, family, and phylum). DA/DP analyses employed (penalized) likelihood ratio, F, or Wald tests as appropriate to test the significance of the endoscopy outcome as a whole as well as for the post-hoc tests across endoscopy outcome groups. Since a consensus approach is currently recommended, we considered taxa to be differentially abundant/prevalent if detected by at least two independent methods [30].

Primary analyses were either marginal or adjusted by center. Sensitivity analyses included further covariate adjustment (proton pump inhibitors use, smoking status, alcohol status, and presence of symptoms) as well as stratification by center. Missing covariate data were handled with multivariate imputation by chained equations using the mice package (v. 3.15.0) [31]. Since the frequency of missing data was less than 1% for all but one covariate (presence of symptoms, 8%), sensitivity analyses were conducted with averaged imputed data as well as with complete case data. Further multiplicity adjustment was also performed to assess the robustness of potentially significant results, given the multiple tools and taxonomic ranks used for the primary analyses. Data and code are available at github: https://github.com/biomehub/microbiome-and-gastric-alterations.

## Results

### Characteristics of the participants

A total of 266 samples were prospectively collected. The oral microbiome of 73 individuals in Cohort 1, and 193 individuals in Cohort 2, was characterized. The sociodemographic, lifestyle habits, and clinical findings of the participants, including age, gender, education level, smoking, alcohol use, gastrointestinal symptoms, use of proton pump inhibitors, and upper gastrointestinal endoscopy outcomes are presented in Table 1.

**Table 1:** Sociodemographic, lifestyle habits and clinical findings of the study groups.

	Cohort 1 (n = 73)	Cohort 2 (n = 193)
**Mean age (years)**	56.54 (35–71)	49 (21–87)
**Gender**		
M	33 (45.2%)	93 (48.18%)
F	39 (53.42%)	99 (51.29%)
No information	1 (1.38%)	1 (0.53%)
**Education level**		
Illiteracy	37 (50.68%)	0 (0.00%)
Elementary School	23 (31.50%)	1 (0.52%)
High School	8 (10.95%)	15 (7.77%)
University	5 (6.84%)	170 (88.08%)
Not answered	0 (0.00%)	7 (3.63%)
**Smoking**		
Yes	9 (12.33%)	8 (4.15%)
No	64 (87.67%)	183 (94.82%)
Not answered	0 (0.00%)	2 (1.03%)
**Alcohol use**		
Yes	9 (12.33%)	38 (19.69%)
No	64 (87.67%)	135 (69.95%)
Not answered	0 (0.00%)	20 (10.36%)
**Gastrointestinal symptoms**		
Yes	25 (34.25%)	132 (68.4%)
No	48 (65.75%)	60 (31.08%)
Not answered	0 (0.00%)	1 (0.52%)
**Use of proton pump inhibitors**		
Yes	4 (5.48%)	43 (22.27%)
No	69 (94.52%)	149 (77.20%)
Not answered	0 (0.00%)	1 (0.52%)
**Upper gastrointestinal endoscopy outcome**		
Normal	52 (71.23%)	98 (50.78%)
Erosive esophagitis (EE)	4 (5.48%)	55 (28.50%)
Gastroduodenal peptic disease (GPD)	17 (23.29%)	21 (10.88%)
EE + GPD	0 (0.00%)	19 (9.84%)

The mean age was 56.54 years in Cohort 1 and 49 years in Cohort 2. There was no difference regarding gender distribution, smoking, and alcohol use in both cohorts. However, there was a remarkable disparity in education level, mainly due to the lower income for participants in Cohort 1. Considering the prevalence of symptoms and the use of proton pump inhibitors, Cohort 1 had fewer symptomatic patients.

### Description of the oral microbiome composition

**Fig 1:**
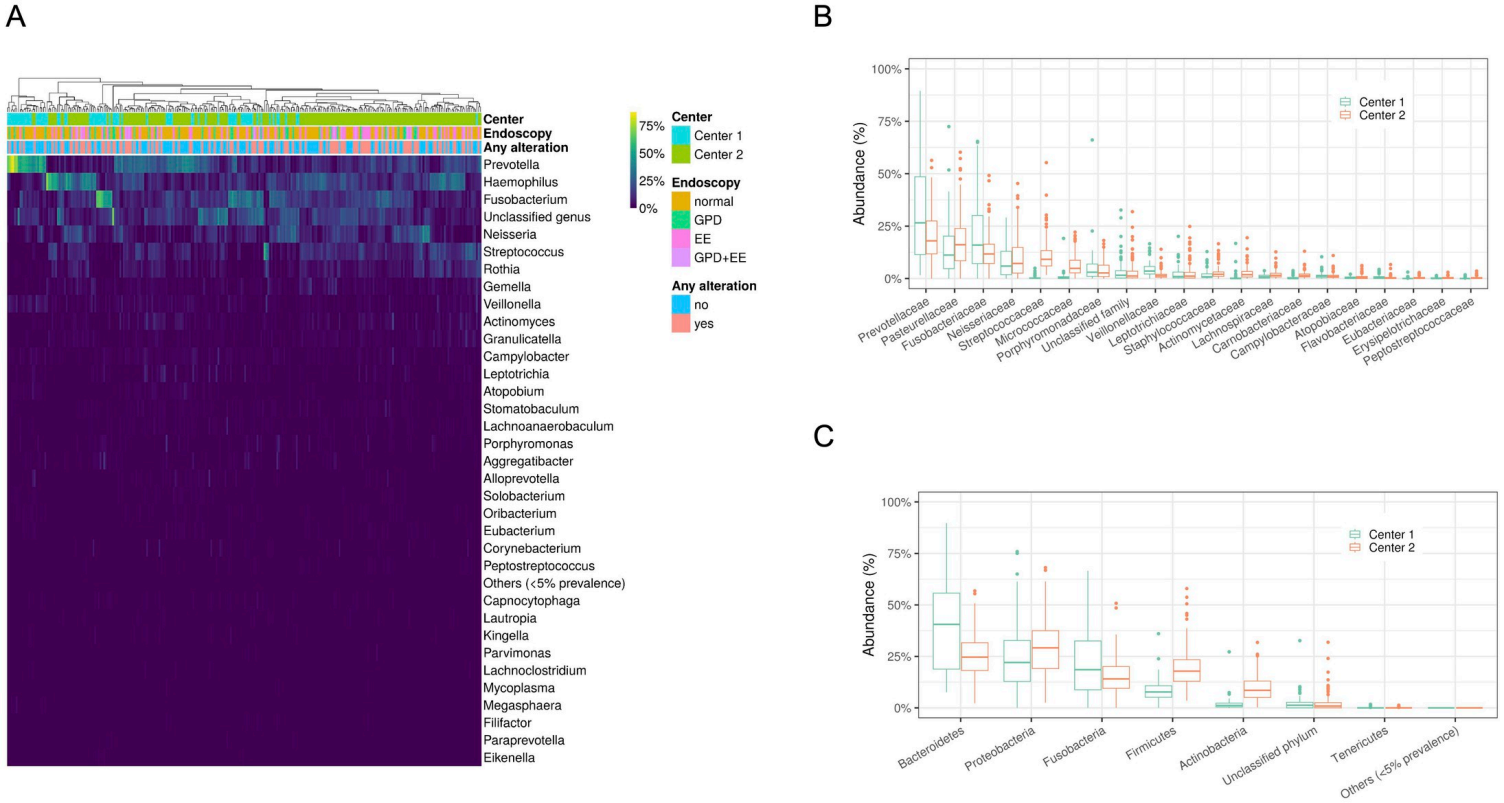
Characterization of the oral microbiome through 16S amplicon sequencing. Heat map of relative abundance of genera present in at least 5% of all samples (A). Box plot of the twenty most abundant families (B) and phyla (C) by center.

The overall composition of the oral microbiome as assessed by 16S amplicon sequencing is depicted in Fig 1. The relative abundance for all genera presents in at least 5% of all samples (14 of 266) ranked by overall average abundance is shown in Fig 1A. The five most abundant classified genera included *Prevotella*, *Haemophilus*, *Fusobacterium*, *Neisseria*, and *Streptococcus*. The average abundance of unclassified sequences at the genus level was 13.4% (95% CI, 12.3% to 14.6%).

The five most abundant families correspond closely to the most abundant genera (Fig 1B). Unclassified sequences were much less abundant on average at the family level: 3.2% (95% CI, 2.6% to 3.8%). Abundance distribution was similar between centers except for the Streptococcaceae and Micrococcaceae families. At the phylum level, the most abundant taxa were Bacteroidetes, Proteobacteria, Fusobacteria, Firmicutes, and Actinobacteria. Center 2 showed substantially higher abundance of Firmicutes and Actinobacteria as compared to Center 1 (Wilcoxon rank sum test p < 10^−16^ for both), while Center 1 showed slightly higher Bacteroidetes (Wilcoxon rank sum test p < 10^−6^). Other differences were small. Additionaly, we searched for the specific presence of 16S rRNA from *H*. *pylori* in oral samples as the direct causative agent. However, it was only detected in three samples. Aiming to increase the sensitivity for *H*.*pylori* detection, 40 cycles, qPCR-sybr green reactions were performed in oral samples for its specific resistance/virulence genes: vacA, cagE, cagA, tsaA, and ureA, but the results were inconclusive (S1 Table).

### Diversity analysis

**Fig 2:**
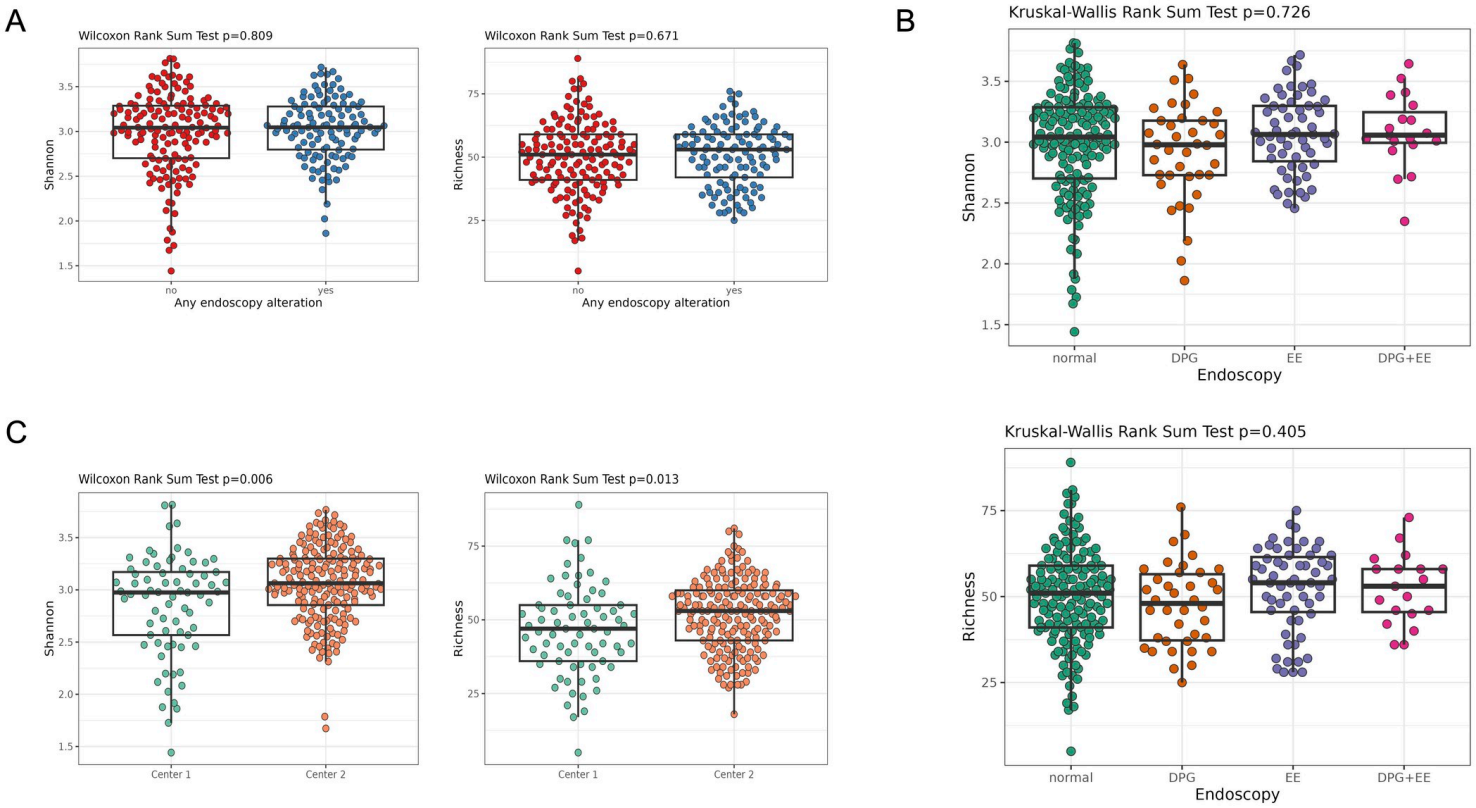
Alpha diversity analysis of oral microbiome samples. Box plots of Richness and Shannon index across patients with or without any endoscopic alteration (A), across endoscopy outcomes subgroups (B), and between centers (C). GPD: Gastroduodenal peptic disease. EE: Erosive esophagitis.

We assessed richness and Shannon indexes as primary metrics of alpha diversity and the results are shown in Fig 2. We investigated whether the alpha diversity differed between patients with or without any endoscopic alteration (Fig 2A) as well as across endoscopy subgroups (Fig 2B). We did not find any association between alpha diversity profiles and endoscopy outcomes. We observed a slightly higher alpha diversity in Center 2 than in Center 1 (Fig 2C). Similar results were obtained with covariate-adjusted analyses as well as analyses stratified by center (S1 Fig).

**Fig 3:**
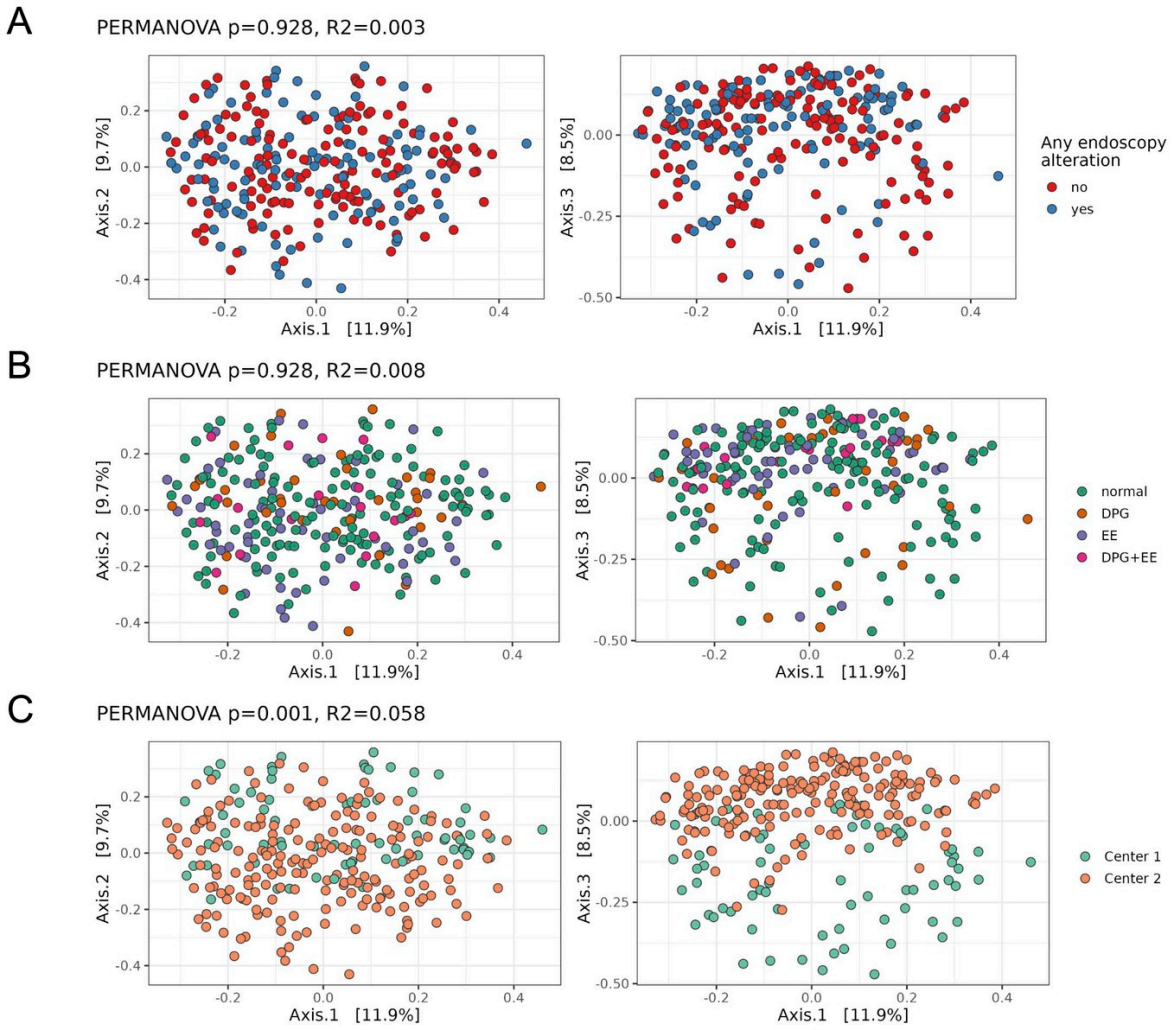
Beta diversity analysis of oral microbiome samples. Principal Coordinate Analysis (PCoA) derived from Bray-Curtis dissimilarities exploring associations between patients with or without any endoscopic alteration (A), across endoscopy outcomes subgroups (B), and between centers (C). GPD: Gastroduodenal peptic disease. EE: Erosive esophagitis.

We also performed Principal Coordinate Analysis to investigate associations between the endoscopy outcomes and the overall oral microbiome profiles regarding beta diversity (Bray-Curtis). Once again, no association with the endoscopy outcomes was detected (Fig 3A and 3B). The second and third coordinates showed clear association with the study populations (Belterra and Sao Paulo), explaining around 6% of the total variance (Fig 3C). These results were consistent with adjusted analyses as well as with analyses stratified by center (S2 Fig).

### Differential abundance analysis

**Fig 4:**
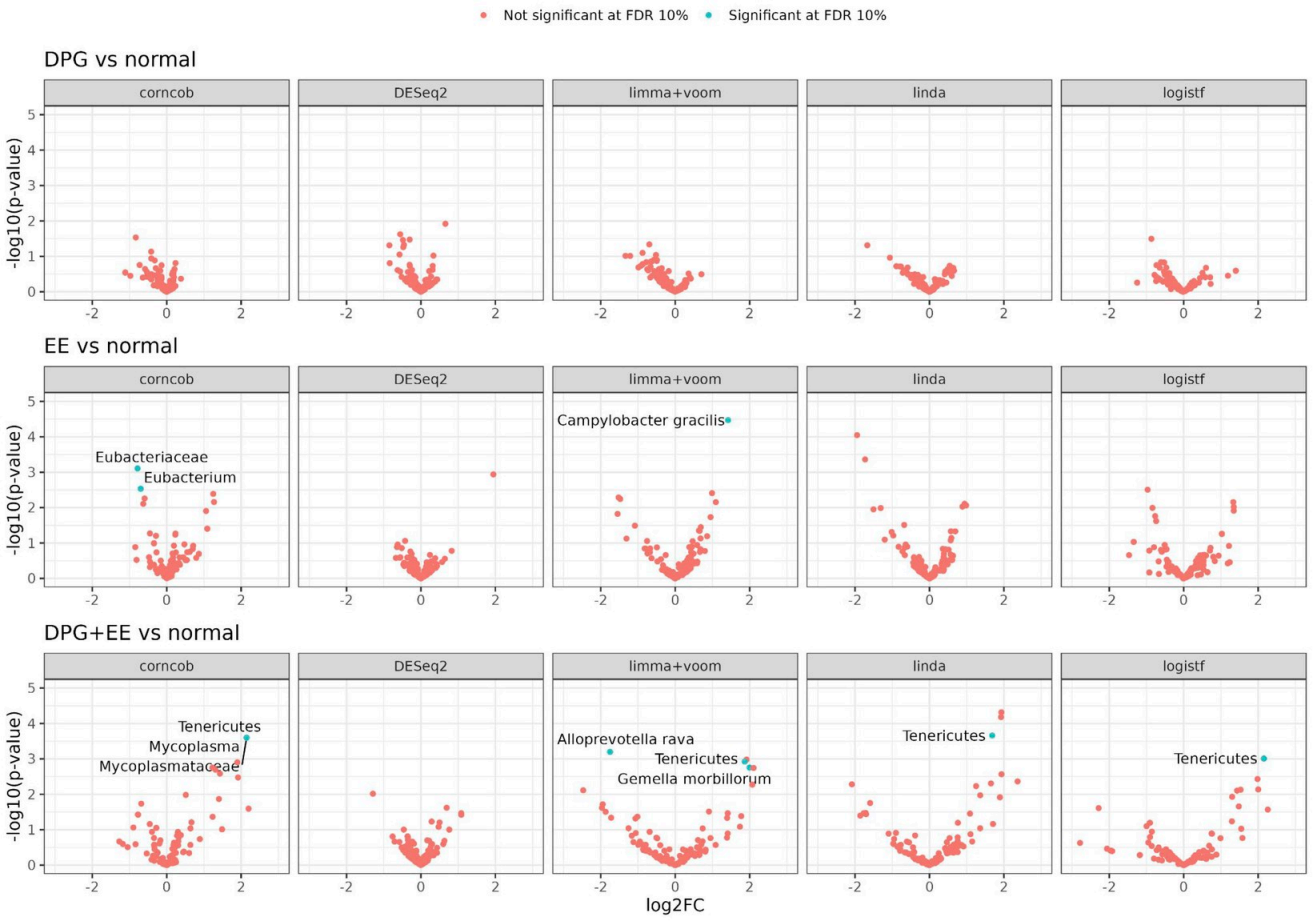
Association between bacterial abundance and endoscopy outcomes based on differential abundance and differential prevalence. GPD: Gastroduodenal peptic disease. EE: Erosive esophagitis.

The association between bacterial abundance and endoscopy outcomes was assessed using a consensus approach based on four differential abundance (DA) methods as well as one differential prevalence (DP) method (see Methods). This consensus approach was chosen as DA is known to generate inconsistent results[30]. DP analysis was employed to assess variation in presence/absence of taxa across the studied groups. At the same time, the four DA tools were chosen to cover a broad range of statistical approaches. The results are shown in Fig 4.

**Fig 5:**
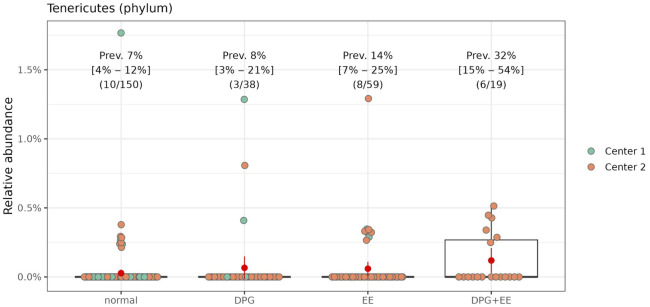
Relationship between abundance of the Tenericutes phylum and endoscopy outcomes. Box plots of the relative abundance of Tenericutes between patients with or without (“normal”) endoscopic alteration, by center. GPD: Gastroduodenal peptic disease. EE: Erosive esophagitis.

Confirming the diversity analysis, we observed no major differences in the oral microbiome composition across the endoscopy outcomes: while some taxa were detected by a single statistical method, no taxa were consistently detected by all methods. An increase in the abundance/prevalence of the phylum Tenericutes was detected by four methods (all but DESeq2) in the GPD+EE group as compared to controls, even after taking into account the additional multiplicity due to using multiple DA/DP tools (Fig 5). However, this result is not to be overinterpreted as the GPD+EE group contains only 19 participants, all from the Cohort 2. Moreover, the statistical significance disappears fromall methods once the multiplicity adjustment accounts for the multiple taxonomic rankings tested. Given the apparently weak signal and extensive data exploration, any hope of clinical importance of this difference requires careful validation of external data.

## Discussion

Belterra and Sao Paulo, two Brazilian cities, exhibit marked differences. According to the Brazilian Institute of Geography and Statistics (IBGE), Belterra has a population of 18,099, whereas Sao Paulo is home to 11,451,245 people. Belterra’s expansive territory results in a low population density of 4.11 individuals per square kilometer, in contrast to Sao Paulo’s 7,527.76. The Human Development Index (HDI) reveals significant disparities, with Belterra at 0.588 and Sao Paulo at 0.805, indicating variations in development. Economically, Belterra’s Gross Domestic Product (GDP) per capita is R$10,460.11, significantly less than Sao Paulo’s R$60,750.09 [32,33]. These differences in population, geography, HDI, and GDP per capita contribute to unique socio-economic structures, which, in turn, influence the experiences and opportunities available to residents of these two cities.

Despite being conducted among the European population, the study performed by Paliova and colleagues (2019) [34] highlights a significant connection between a higher Human Development Index (HDI) and increased years of schooling. This correlation could explain the disparities in educational levels between the two cohorts evaluated in this study.

A low Human Development Index (HDI) and low education levels are usually connected to poorer health status [35,36]. For instance, communities or regions with lower HDI scores typically face challenges in establishing and maintaining healthcare infrastructure, reducing access to medical facilities, professionals, and essential treatments. Consequently, these can result in inadequate healthcare outcomes and an overall lower quality of life for the population. In this study, we observed that in Cohort 1 (Belterra), only 16% of participants with symptoms use proton pump inhibitors, while in Cohort 2 (São Paulo), 32.6% of participants with symptoms use these medications. This discrepancy may be attributed to limited access to healthcare services and to lower educational levels in Belterra’s population, potentially resulting in inadequate healthcare treatment.

Despite sociodemographic differences between the two cohorts, this study’s genera, families, and phyla profiles aligned with anticipated expectations. For instance, the higher presence of genera such as *Prevotella*, *Haemophilus*, *Fusobacterium*, *Neisseria*, and *Streptococcus* was already observed in previous studies [37,38].

Although the oral microbiota has been explored as a diagnostic method for various cancers, including oral squamous cell carcinoma[39,40], pancreatic cancer [41], and lung cancer [42,43], the current research does not provide sufficient evidence to establish the oral microbiome as a biomarker for gastric diseases, as no significant correlation between microbial patterns and gastric alterations was observed.

A noteworthy finding in the current study was the identification of the Tenericutes phylum in individuals diagnosed with erosive esophagitis combined with gastroduodenal peptic disease (GPD+EE). Intriguingly, a study conducted in 2014 detected Tenericutes in small quantities in salivary samples from patients with oral squamous cell carcinoma, while no presence of Tenericutes was observed in corresponding control samples. This finding suggests the potential of Tenericutes as a biomarker for oral cancer [44]. However, despite this connection, the presence of Tenericutes in our present study does not supply robust enough evidence to label it as a biomarker for gastric disease. An important limitation of this analysis lies in the small sample size, involving only 19 subjects with GPD+EE from a single cohort, restricting its representativeness for broader generalization.

The most significant difference observed in this study was the oral microbiome profile between the two cohorts. Although the primary aim of this study was to evaluate a broad diagnostic test for gastric alterations, the heterogeneity of the study population also represented a significant limitation. Variations in oral health status[45,46], comorbidities[45], and ongoing antibiotic treatments [47] are potential confounding factors that could influence the oral microbiome profile and, consequently, the results observed.

This observation underscores the need for a more extensive study to address the influence of population stratification, especially when aiming to develop a diagnostic test with widespread applicability. Without such studies, it may be necessary to identify disease markers specific to each population or their distinctive microbiome features. Among the two populations analyzed in the present study, there was no indication that the oral microbiome can be a valuable tool for directly detecting *H*. *Pylori* or generating diagnostic markers for evaluating gastric health. So, it may be worthwhile to consider an analysis refinement by categorizing populations into subgroups based on factors like disease grade or other specific disease criteria.

## Conclusion

While identifying Tenericutes in erosive esophagitis with gastroduodenal peptic disease was intriguing, the data generated by this study did not establish the oral microbiome composition as a reliable marker for gastric health. Moreover, the significant differences in oral microbiome profiles highlight the need for broader studies in developing non-invasive diagnostic tests for gastric diseases.

## Supporting information

S1 Fig Alpha diversity analysis stratified by center. Top panels show Shannon diversity index for center 1 (A) and center 2 (B). Bottom panels show richness for center 1 (C) and center 2 (D).
(TIF)

S2 Fig Beta diversity analysis stratified by center. Top panels show Principal Coordinate Analysis (PCoA) for center 1 (A), while bottom panel shows PCoA for center 2 (B). All PCoAs were based on Bray-Curtis dissimilarities.
(TIF)

S1 Table H.pylori detection sensitivity. qPCR-sybr green reactions were performed in oral samples for specific resistance/virulence genes: vacA, cagE, cagA, tsaA and ureA.
(DOCX)
