# Supplementary figures and images for: Oral microbiome test as an alternative diagnostic tool for gastric alterations: A prospective, bicentric cross-sectional study
Source: PLoS One. 2024 Dec 2;19(12):e0314660. doi: 10.1371/journal.pone.0314660 (PMC11611075; doi:10.1371/journal.pone.0314660)

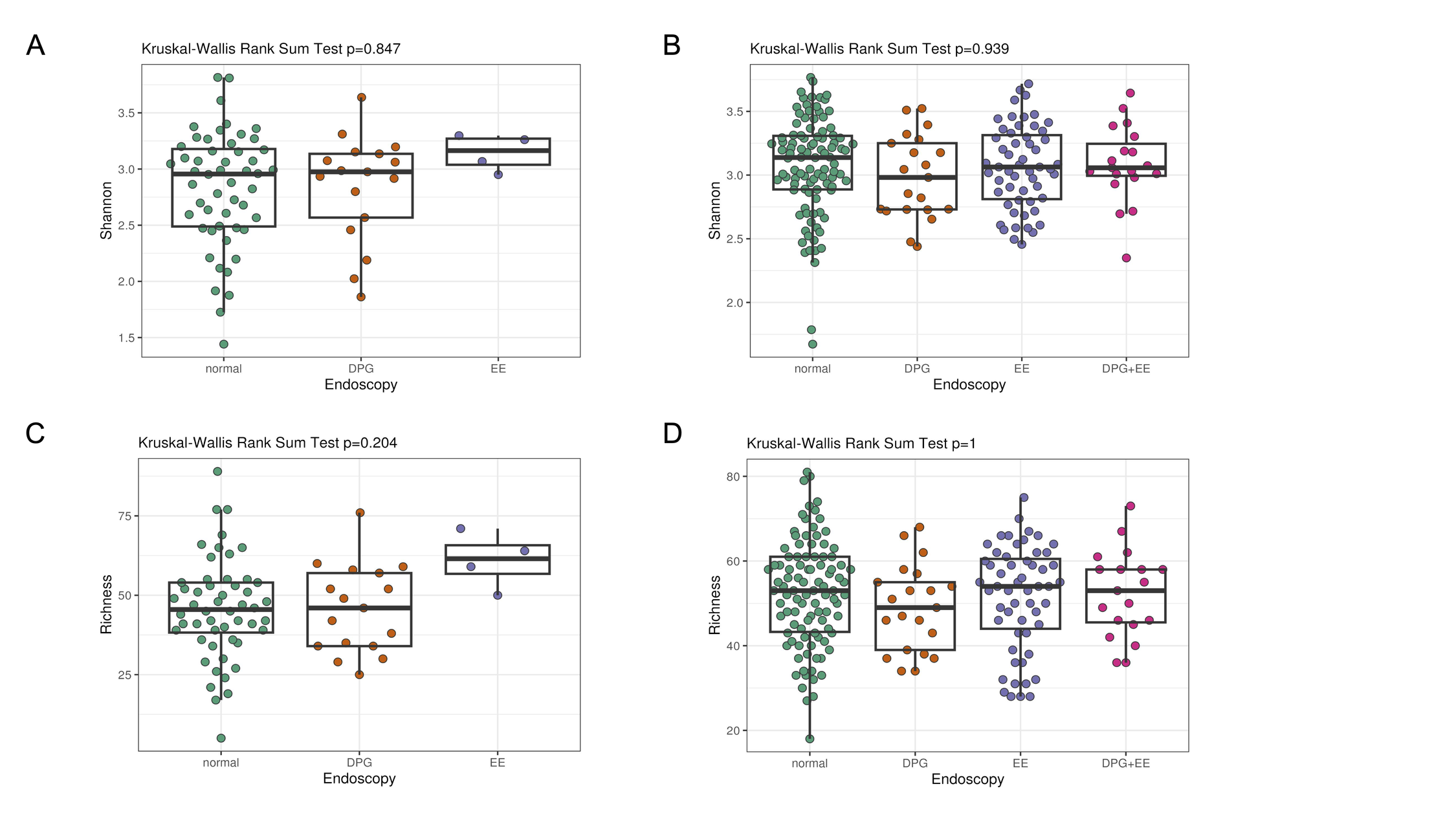

Supplement: S1 Fig — Top panels show Shannon diversity index for center 1 (A) and center 2 (B). Bottom panels show richness for center 1 (C) and center 2 (D). (TIF) [file pone.0314660.s001.tif]

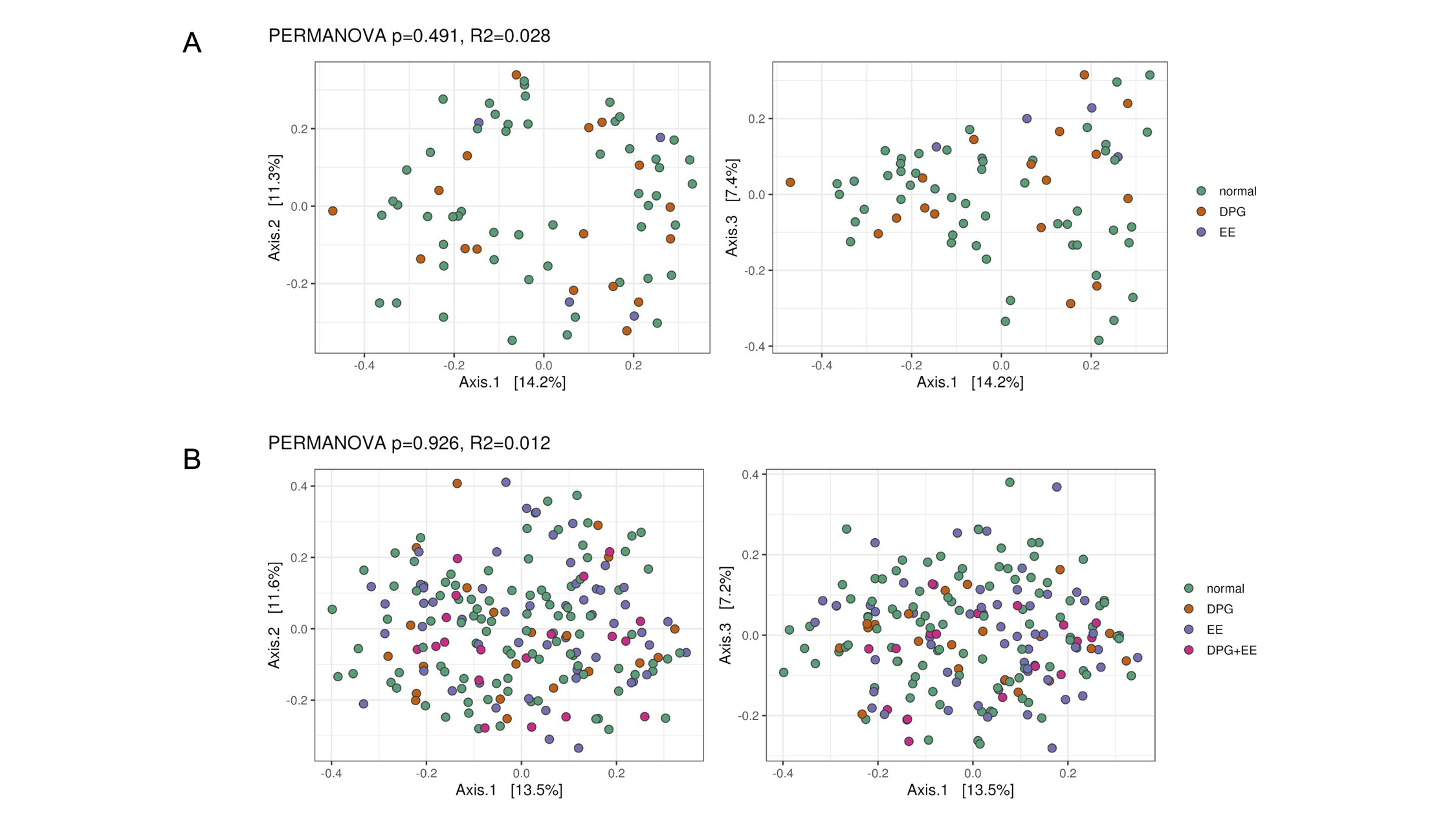

Supplement: S2 Fig — Top panels show Principal Coordinate Analysis (PCoA) for center 1 (A), while bottom panel shows PCoA for center 2 (B). All PCoAs were based on Bray-Curtis dissimilarities. (TIF) [file pone.0314660.s002.tif]
