# Supplementary material for: Oral microbiome test as an alternative diagnostic tool for gastric alterations: A prospective, bicentric cross-sectional study
Source: PLoS One. 2024 Dec 2;19(12):e0314660. doi: 10.1371/journal.pone.0314660 (PMC11611075; doi:10.1371/journal.pone.0314660)
Supplement: S1 Table — qPCR-sybr green reactions were performed in oral samples for specific resistance/virulence genes: vacA, cagE, cagA, tsaA and ureA. (DOCX) [file pone.0314660.s003.docx]

**S1 Table.** qPCR-sybr green reactions performed in oral samples for specific resistance/virulence genes: vacA, cagE, cagA, tsaA and ureA.

| **Sample** | ***H. pylori* 16S rRNA (V3-V4)** | **vacA** | **cagE** | **cagA** | **tsaA** | **ureA** |
| --- | --- | --- | --- | --- | --- | --- |
| 171107015202 | Negative | Negative | Negative | Negative | Negative | Negative |
| 171107015203 | Negative | Negative | Negative | Negative | **Positive** | Negative |
| 171107015204 | Negative | Negative | Negative | Negative | Negative | Negative |
| 171107015205 | **Positive** | **Positive** | **Positive** | **Positive** | **Positive** | Negative |
| 171107015206 | Negative | Negative | Negative | Negative | **Positive** | Negative |
| 171107015207 | Negative | Negative | Negative | Negative | Negative | Negative |
| 171107015208 | Negative | Negative | Negative | Negative | Negative | Negative |
| 171107015209 | Negative | Negative | Negative | Negative | Negative | Negative |
| 171107015211 | Negative | Negative | Negative | Negative | Negative | Negative |
| 171107015212 | Negative | Negative | Negative | Negative | **Positive** | Negative |
| 171107015213 | Negative | Negative | Negative | Negative | **Positive** | Negative |
| 171107015214 | Negative | Negative | Negative | Negative | Negative | Negative |
| 171107015215 | Negative | Negative | Negative | Negative | **Positive** | Negative |
| 171107015216 | Negative | Negative | Negative | Negative | Negative | Negative |
| 171107015217 | Negative | Negative | Negative | Negative | **Positive** | Negative |
| 171107015218 | Negative | Negative | Negative | Negative | **Positive** | Negative |
| 171107015219 | Negative | Negative | Negative | Negative | **Positive** | Negative |
| 171107015220 | Negative | Negative | Negative | Negative | Negative | Negative |
| 171107015221 | Negative | Negative | Negative | Negative | Negative | Negative |
| 171107015222 | Negative | Negative | Negative | Negative | **Positive** | Negative |
| 171107015223 | Negative | Negative | Negative | Negative | **Positive** | Negative |
| 171107015224 | Negative | **Positive** | Negative | Negative | **Positive** | Negative |
| 171107015225 | Negative | Negative | Negative | Negative | **Positive** | Negative |
| 171107015226 | Negative | Negative | Negative | Negative | Negative | Negative |
| 171107015227 | Negative | Negative | Negative | Negative | Negative | Negative |
| 171107015228 | Negative | Negative | Negative | Negative | **Positive** | Negative |
| 171107015229 | Negative | Negative | Negative | Negative | **Positive** | Negative |
| 171107015230 | Negative | Negative | Negative | Negative | Negative | Negative |
| 171107015231 | Negative | Negative | Negative | Negative | **Positive** | Negative |
| 171107015232 | Negative | Negative | Negative | Negative | **Positive** | Negative |
| 171107015233 | Negative | Negative | Negative | Negative | **Positive** | Negative |
| 171107015234 | Negative | Negative | Negative | Negative | Negative | Negative |
| 171107015235 | **Positive** | **Positive** | **Positive** | **Positive** | **Positive** | Negative |
| 171107015236 | Negative | Negative | Negative | Negative | **Positive** | Negative |
| 171107015237 | Negative | Negative | Negative | Negative | Negative | Negative |
| 171107015238 | Negative | Negative | Negative | Negative | **Positive** | Negative |
| 171107015239 | Negative | Negative | Negative | Negative | **Positive** | Negative |
| 171107015240 | Negative | Negative | Negative | Negative | **Positive** | Negative |
| 171107015242 | Negative | Negative | Negative | Negative | Negative | Negative |
| 171107015243 | Negative | Negative | Negative | Negative | Negative | Negative |
| 171107015244 | Negative | Negative | Negative | Negative | Negative | Negative |
| 171107015245 | Negative | Negative | Negative | Negative | Negative | Negative |
| 171107015247 | Negative | Negative | Negative | Negative | Negative | Negative |
| 171107015248 | Negative | Negative | Negative | Negative | Negative | Negative |
| 171107015249 | Negative | Negative | Negative | Negative | Negative | Negative |
| 171107015250 | Negative | Negative | Negative | Negative | Negative | Negative |
| 171107015251 | **Positive** | **Positive** | **Positive** | Negative | **Positive** | Negative |
| 171107015252 | Negative | Negative | Negative | Negative | **Positive** | Negative |
| 171107015253 | Negative | Negative | Negative | Negative | Negative | Negative |
| 171107015254 | Negative | Negative | Negative | Negative | **Positive** | Negative |
| 171107015255 | Negative | Negative | Negative | Negative | Negative | Negative |
| 171107015256 | Negative | Negative | Negative | Negative | Negative | Negative |
| 171107015257 | Negative | Negative | Negative | Negative | **Positive** | Negative |
| 171107015259 | Negative | Negative | Negative | Negative | **Positive** | Negative |
| 171107015260 | Negative | Negative | Negative | Negative | Negative | Negative |
| 171107015261 | Negative | Negative | Negative | Negative | Negative | Negative |
| 171107015262 | Negative | Negative | Negative | Negative | Negative | Negative |
| 171107015263 | Negative | Negative | Negative | Negative | Negative | Negative |
| 171107015264 | Negative | Negative | Negative | Negative | Negative | Negative |
| 171107015265 | Negative | Negative | Negative | Negative | Negative | Negative |
| 171107015266 | Negative | Negative | Negative | Negative | **Positive** | Negative |
| 171107015267 | Negative | Negative | Negative | Negative | **Positive** | Negative |
| 171107015268 | Negative | Negative | Negative | Negative | **Positive** | Negative |
| 171107015269 | Negative | Negative | Negative | Negative | **Positive** | Negative |
| 171107015270 | Negative | Negative | Negative | Negative | **Positive** | Negative |
| 171107015271 | Negative | Negative | Negative | Negative | **Positive** | Negative |
| 171107015273 | Negative | Negative | Negative | Negative | Negative | Negative |
| 171107015274 | Negative | Negative | Negative | Negative | **Positive** | Negative |
| 171107015275 | Negative | Negative | Negative | Negative | Negative | Negative |
| 171107015276 | Negative | Negative | Negative | Negative | **Positive** | Negative |
| 171107015278 | Negative | Negative | Negative | Negative | **Positive** | Negative |
| 171107015279 | Negative | Negative | Negative | Negative | Negative | Negative |
| 171107015280 | Negative | Negative | Negative | Negative | Negative | Negative |
